# Reviewing the state of the art of probiotics as clinical modalities for brain–gut–microbiota axis associated disorders

**DOI:** 10.3389/fmicb.2022.1053958

**Published:** 2022-11-25

**Authors:** Cato Wiegers, Mariët A. Veerman, Robert Jan Brummer, Olaf F. A. Larsen

**Affiliations:** ^1^Athena Institute, Vrije Universiteit Amsterdam, Amsterdam, Netherlands; ^2^Faculty of Medical and Health Sciences, Nutrition-Gut-Brain Interactions Research Centre, School of Medical Sciences, Örebro University, Örebro, Sweden

**Keywords:** probiotics, gut–brain, brain–gut–microbiota axis, mental disorders, neurological disorders, gastrointestinal disorders, state of the art

## Abstract

The rise in prevalence of mental and neurological disorders is causing a high burden on society, however adequate interventions are not always available. The brain–gut–microbiota axis (BGMA) may provide a new angle for the development of clinical modalities. Due to the intricate bi-directional signaling between the brain and the gut, it may be helpful to look into interventions that target the gut, such as probiotics. Therefore, this review aimed to investigate the state of the art of probiotics and their potential as clinical modalities for BGMA-associated indications by gaining insight into patents and clinical trials that have been applied for and executed since 1999. A total of 565 patents and 390 clinical trials were found, focusing on probiotic applications for 83 indications. Since the start of the 21st century, the highest numbers of patents and clinical trials were related to primary neuropsychological, affective (depression, anxiety) and cognitive disorders, neurodegenerative and/or inflammatory brain disorders (Alzheimer’s disease, Parkinson’s disease, amongst others), and gastrointestinal disorders (irritable bowel syndrome). The locations where the most patents and clinical trials were registered included China, the United States, and Iran. From 1999 to ~2013 a slight growth could be seen in the numbers of patents and clinical trials, followed by an almost exponential growth from ~2013 onwards. Overall, the developments of the state of the art were in accordance with previous research, however it appeared that clinical trials showed a slightly slower growth compared to patents, which may have implications for the future implementation of probiotics as clinical modalities for BGMA-associated indications.

## Introduction

In our ageing population, neurodegenerative diseases such as Alzheimer’s and Parkinson’s disease are becoming more prevalent (Hou et al., 2019) Inflammatory disorders of the gut are associated with neuroinflammatory changes in the brain (Sanmarco et al., 2021), earlier onset of dementia (Zhang et al., 2021) and infer an increased risk of Parkinson’s disease (Villumsen et al., 2019). In addition, the World Health Organization (WHO) has stated that mental indications are the leading cause of disability and illness among adolescents between 10 and 19 years old (World Health Organization, 2021a). The COVID-19 pandemic of the past few years is another cause of concern, as its detrimental effects are not limited to physical health, but also impact mental health. Due to the high burden of mental and neurological disorders, adequate therapies are needed. However, currently used treatment plans are often not effective or not available at all (Thorpe et al., 2021). An additional problem is the rising cost of (mental) health care (World Health Organization, 2021b). Because of this, a global shift is happening from treatment focused care to a more preventive approach (Globe Newswire, 2020). Moreover, according to Larsen and van de Burgwal (2021), it is important to focus on health not only from the individual perspective but also from a microbiological and environmental perspective. This means that regarding mental and neurological disorders, it is valuable to include the microbiota when researching possible clinical modalities.

A large body of evidence shows that the brain is inextricably linked with the gut and the gut microbiota present therein. This is referred to as the brain-gut-microbiota axis (BGMA) and comprises various ways of communication between the gut microbiota, the gut, and the brain, through neurological, endocrine, and immune pathways (Liu and Zhu, 2018). The BGMA is described as dynamic and bidirectional and signaling occurs directly and indirectly between the central and enteric nervous systems (Arneth, 2018; Morais et al., 2021). An example of BGMA signaling includes the production of neurotransmitters and metabolites such as serotonin, noradrenalin, and short-chain fatty acids by the gut microbiota which can interact with the immune system *via* blood circulation or direct signaling to the brain *via* the enteric nervous system and the vagus nerve (Morais et al., 2021). Additionally, the brain can influence the gut *via* the hypothalamic–pituitary–adrenal (HPA) axis, which is activated by stress and triggers the production of cortisol, in turn influencing the permeability of the gut (Morais et al., 2021).

Based on this knowledge, it has been found that several mental and neurological disorders are linked to the BGMA. For example, autism spectrum disorder (ASD; Cryan and Dinan, 2012; Silva et al., 2020), Parkinson’s disease (Sampson et al., 2016; Silva et al., 2020), and anxiety states and (severity of) depressions (Bercik et al., 2010; Chang et al., 2022; Góralczyk-Bińkowska et al., 2022). Besides, patients suffering from a functional bowel disorder are more likely to simultaneously suffer from depression and/or anxieties (Martin et al., 2018). Irritable bowel syndrome (IBS) has also been characterized as a BGMA-associated disorder (Chong et al., 2019).

Because of the clear link between the gut microbiota and the brain, probiotics provide a plausible interventional modality for psychiatric and neurological disorders (Hutchinson et al., 2020; Palepu and Dandekar, 2022). Probiotics are defined as “live microorganisms that, when administered in adequate amounts, confer a health benefit on the host” (Hill et al., 2014, p. 507). Due to promising results, probiotics in the context of mental and neurological indications such as Alzheimer’s disease, ASD, anxiety, and depression have gained more attention (Ansari et al., 2020). Additionally, administration of various types of probiotics has demonstrated to significantly decrease the severity of IBS symptoms (Chong et al., 2019; Skrzydło-Radomańska et al., 2021; van der Geest et al., 2022a,b). Recently, a 4-week administration with a multi-strain probiotic showed changes in brain activity and connectivity (Edebol Carlman et al., 2022; Rode et al., 2022). The rise in popularity of probiotics as clinical modalities for BGMA-related indications is further emphasized by the large number of products that have become available on the market. Moreover, the highly specific term psychobiotics refers to probiotics (and prebiotics) that when administered to the host, have beneficial effects on mental health by interaction with the gut microbiome (Sarkar et al., 2016).

However, the state of the art regarding probiotic applications for BGMA-associated disorders remains unknown. Therefore, the aim of this review is to close this gap by investigating patents and clinical trials since the start of the 21st century. Patents can be used to indicate market trends and because they are valid for 20 years, they give insight into long-term research developments (Feddema and Claassen, 2018; Janse et al., 2020). Clinical trials are part of a later stage in the research process and provide insight into short-term trends and developments (Ramezanpour et al., 2015; Janse et al., 2020). Together, patents and clinical trials provide a comprehensive scope of the research landscape, which can be used to estimate and describe the state of the art.

## Materials and methods

This systematic review was designed to provide insight into the state of the art of probiotics and their potential to treat, prevent or alleviate symptoms of BGMA-related disorders. Firstly, a list of relevant mental, neurological, and gastrointestinal indications was formulated. Secondly, several patent and clinical trial databases were queried according to methods used in previous research (Ramezanpour et al., 2015; Janse et al., 2020; Neevel et al., 2020; Wiegers et al., 2022). For a schematic overview of the workflow, see Supplementary Figure 1.

### Selection of BGMA-associated indications

To demarcate the indications included in this study, a first orientation in scientific literature was carried out. Based on the 5th edition of the Diagnostic and Statistical Manual of Mental Disorders (DSM-5) of the American Psychiatric Association (2013), MedlinePlus of the US National Library of Medicine (2016, 2021), Duke University Health System (2022), and several reviews (Umbrello and Esposito, 2016; Kim and Shin, 2018; Suganya and Koo, 2020; Morais et al., 2021), a draft list of mental and neurological indications was formulated. The final product (Supplementary Table 1) consisted of a table with all indications (*n* = 137) that were investigated in this review.

### Data collection and selection

For each indication, search queries were formulated to be used in 4 different databases: the European Patent Office (EPO) Espacenet, the US NLM ClinicalTrials.gov (CT.gov), the European Medicines Agency (EMA) EU Clinical Trials Registry (CTR.eu), and the WHO International Clinical Trials Registry Platform (ICTRP). The Espacenet database was selected as it provides original documents of over 130 million patents, dating back as far as 1782 (European Patent Office, 2022). The CT.gov database was selected because it contains over 400,000 clinical trial records from 220 countries (ClinicalTrials.gov, 2021). The CTR.eu database contains all clinical trial records that have been entered in the EU clinical trials database (EudraCT). Clinical trials from this database are also included in the ICTRP, which contains data from 17 different data providers covering over 200 countries (World Health Organization, 2021c). Combining data from all three clinical trial databases ensured a global scope where no relevant clinical trials could have been left out.

Searches were specified to retrieve results regarding the selected indication as a target of a probiotic intervention, which was achieved through the inclusion of key words relating to probiotics. An example of a final search query used in the 3 clinical trial databases is as follows: (“ALS” OR “amyotrophic lateral sclerosis”) AND (“Probiotic” OR “Probiotics” OR “*Lactobacillus*” OR “*Streptococcus*” OR “*Bifidobacterium*” OR “*Enterococcus*” OR “*Escherichia*” OR “*E. coli*” OR “*Saccharomyces*” OR “Lactic acid bacterium”). Two keywords were dedicated to *Escherichia coli*, as this is the species of Escherichia bacteria that is most commonly used as a probiotic (Fijan, 2014). Wildcard operators (*) were not used, as they negatively affected the accuracy of search results. For the patent database, several International Patent Classification (IPC) and Cooperative Patent Classification (CPC) codes relating to probiotics were used instead of key words (Espacenet, 2019a). The IPC and CPC systems have been developed to describe the subject of a patent in great detail to allow for more targeted searches without the need for different key words (Espacenet, 2019b). Search strings were optimized under guidance of experts from the Netherlands Enterprise Agency (RVO; Table 1).

**Table 1:** Overview of included patent classification codes.

CPC/IPC code	Description espacenet	
A61K2035/115		“Probiotics”
A61K35/741		“Probiotics (probiotic yeast, e.g., *Saccharomyces*)”
	A61K35/742	“Spore-forming bacteria, e.g., *Bacillus coagulans*, *Bacillus subtilis*, *Clostridium* or *Lactobacillus sporogenes*”
	A61K35/744	“Lactic acid bacteria, e.g., Enterococci, Pediococci, Lactococci, Streptococci or Leuconostocs”
	A61K35/745	“Bifidobacteria”
	A61K35/747	“Lactobacilli, e.g., *L. acidophilus* or *L. brevis*”
A23L33/135		“Bacteria or derivatives thereof, e.g., Probiotics”

Once searches were completed for each indication in all four included databases, patents and clinical trials were screened in twofold to assess their relevance and suitability for this study. Several concrete inclusion criteria were formulated to support this process:

- Studies published from 1999-01-01 to 2022-03-01.
- Studies containing a description of live probiotics only (no killed microorganisms or derivatives thereof).
- Studies relating to the treatment, prevention, or alleviation of symptoms of mental, neurological, or gastrointestinal disorders.
- Studies in gastrointestinal disorders: being related to a mental or neurological disorder.
- Regarding patents: not solely describing a production method or technology.
- Regarding clinical trials: following an interventional study design.

Preliminary searches showed little to no results regarding probiotics and the brain dating back earlier than the 21st century. This justified the time frame of 1999–2022, which was chosen to cover the first decades of the 21st century as well as to compensate for the 18-month application period during which patents are filed, but not yet published. The criterium for clinical trials to follow an interventional study design was set to only include randomized controlled trials, which are considered the standard experimental design for studying effectiveness of an intervention (Hariton and Locascio, 2018). Additionally, a filter was used in Espacenet to exclude veterinary patents, for example relating to animal fodder.

### Data analysis

Patents and clinical trials that met all inclusion criteria were screened for duplicates and analyzed in Microsoft Excel. Since the goal was to create an overview of the state of the art, the focal points of analysis were the indication types, publishing dates, locations, and applicant/sponsor types. Data on the indications were readily stated in the respective patent documents and clinical trial entries, and for both types of data the earliest priority or registry dates were analyzed in a similar fashion. Columns containing these dates were split in Excel to retrieve the year, and filters were applied to count the number of documents that were registered per year. Regarding locations, different methods were used for patent and clinical trial data. For patents, each document code listed either as the primary code or under “also published as” was investigated, as these codes provide information on the country or organization that the document was filed in. For clinical trials, the country in which each trial was executed was selected as the location. Applicant and sponsor types (academic, industrial, governmental, individual or a collaboration between two types) were manually analyzed and color coded based on the provided applicant names, apart from some clinical trials where the sponsor type was explicitly stated in the trial entry.

## Results

### Data inclusion

**Figure 1:**
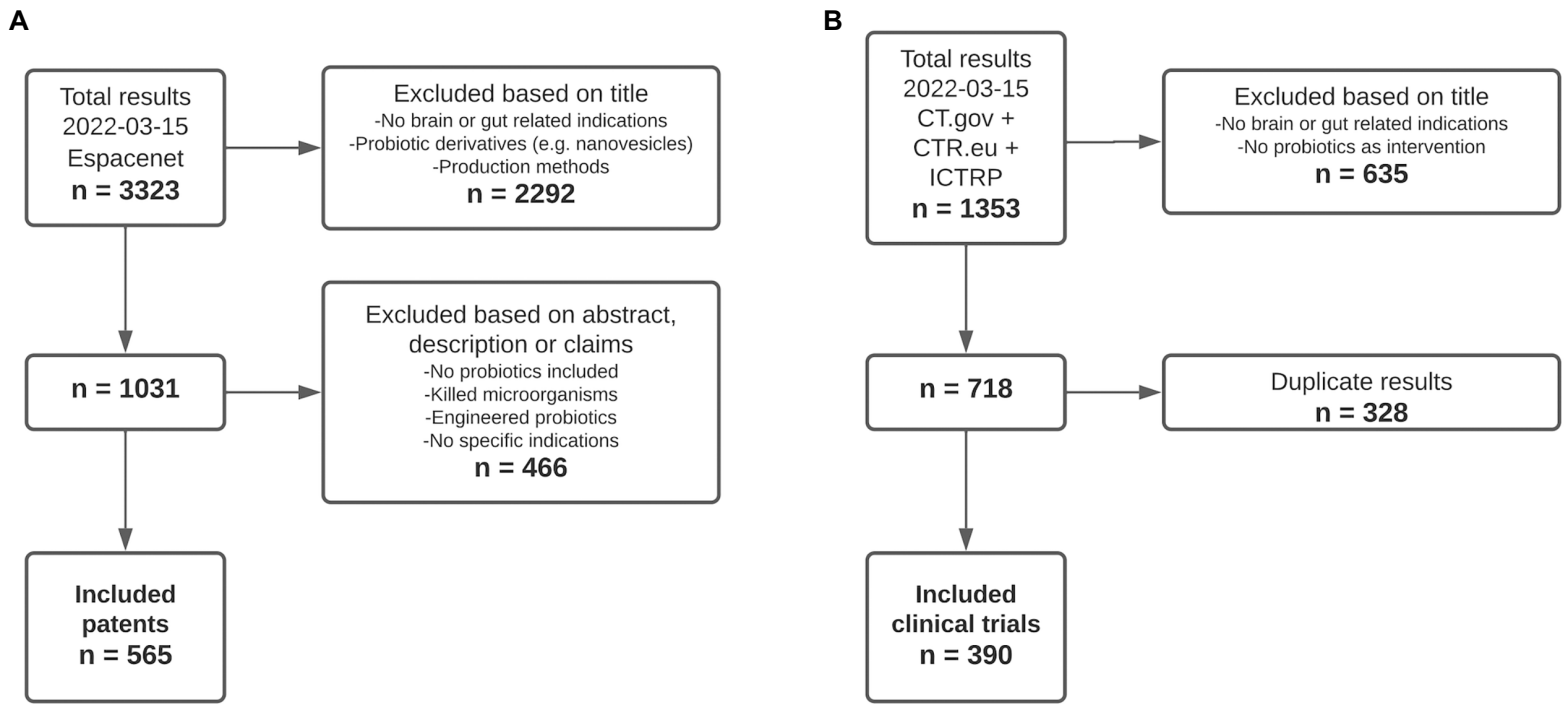
Flowchart showing the inclusion of patents and clinical trials. **(A)** Inclusion process of patent documents. **(B)** Inclusion process of clinical trial records.

After applying the inclusion criteria to all retrieved patent and clinical trial records, a total of 565 patents (Figure 1A) and 390 clinical trials (Figure 1B) were included for further analysis. Main reasons for exclusion of both patents and clinical trials included the descriptions of diseases/disorders that were decided to not be included in this study, e.g., infectious diseases and cancer, as well as interventions that did not include any form of probiotic administration.

### Brain–gut–microbiota axis-associated indications

**Figure 2:**
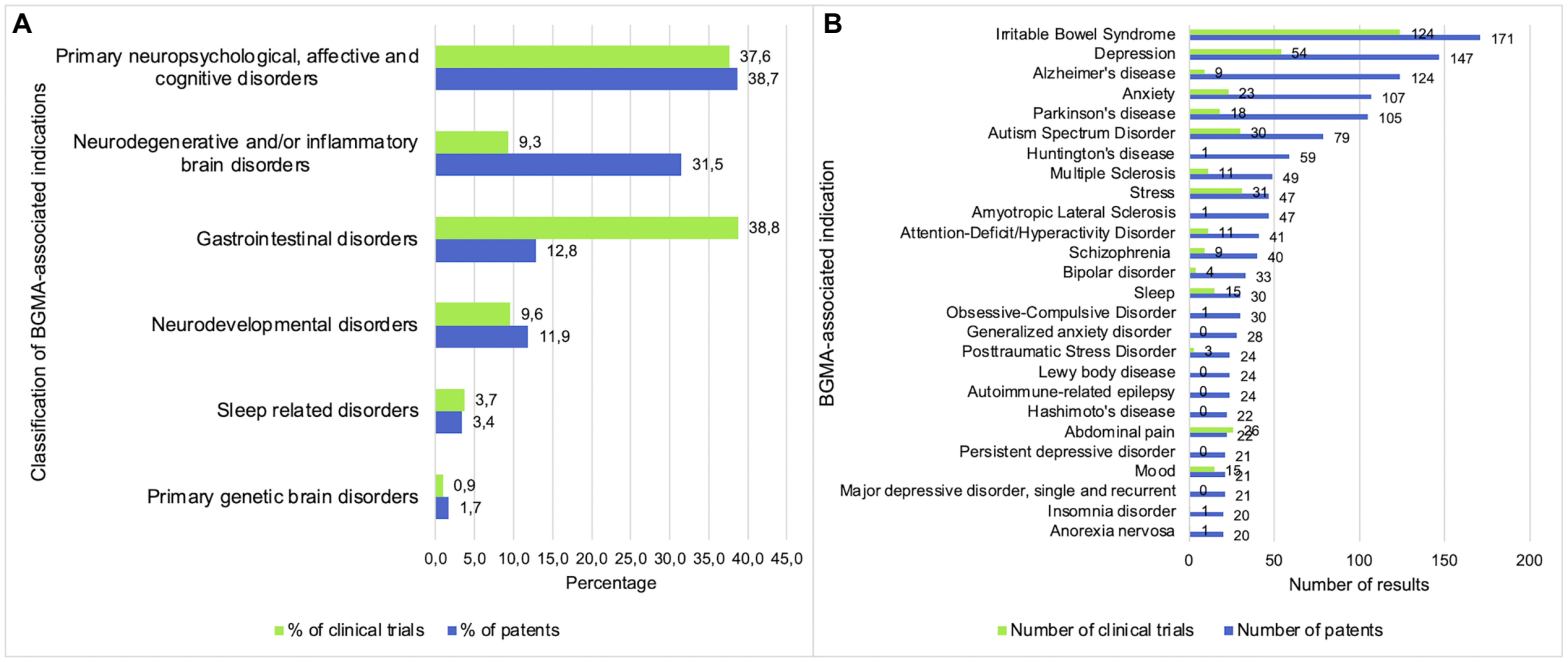
Primary neuropsychological, affective and cognitive disorders are the main focus of patents and clinical trials. **(A)** Percentage of patents and clinical trials per indication category. **(B)** Number of patents and clinical trials per indication.

Out of the 137 indications that were included in the database searches, 83 retrieved one or more patents or clinical trials. Regarding the number of patents and clinical trials that were found for each type of BGMA related indication, Figure 2A shows that the highest percentage of patents were related to primary neuropsychological, affective and cognitive disorders (38.7%) and neurodegenerative and/or inflammatory brain disorders (31.5%). Clinical trials were mainly related to gastrointestinal disorders (38.8%) followed by primary neuropsychological, affective and cognitive disorders (37.6%). For both patents and clinical trials, the lowest percentages were related to sleep disorders (3.4 and 3.7%) and primary genetic brain disorders (1.7 and 0.9%). Considering each indication separately as shown in Figure 2B, the overall highest numbers of both patents (*n* = 171) and clinical trials (*n* = 124) were related to IBS (irritable bowel syndrome). This was followed by depression (all types; *n* = 147 and *n* = 54 respectively), anxiety (*n* = 107 and *n* = 23 respectively) and Parkinson’s disease (*n* = 105 and *n* = 18 respectively). Some indications showed a relatively high discrepancy between the number of patents and clinical trials, including Alzheimer’s disease (*n* = 124 and *n* = 9 respectively), Huntington’s disease (*n* = 59 and *n* = 1 respectively) and ALS (amyotrophic lateral sclerosis; *n* = 47 and *n* = 1 respectively) whereas other indications showed more similar numbers of patents and clinical trials, such as stress (*n* = 47 and *n* = 31 respectively), abdominal pain (*n* = 22 and *n* = 26 respectively) and mood (*n* = 21 and *n* = 15 respectively).

### Chronological and geographical trends

**Figure 3:**
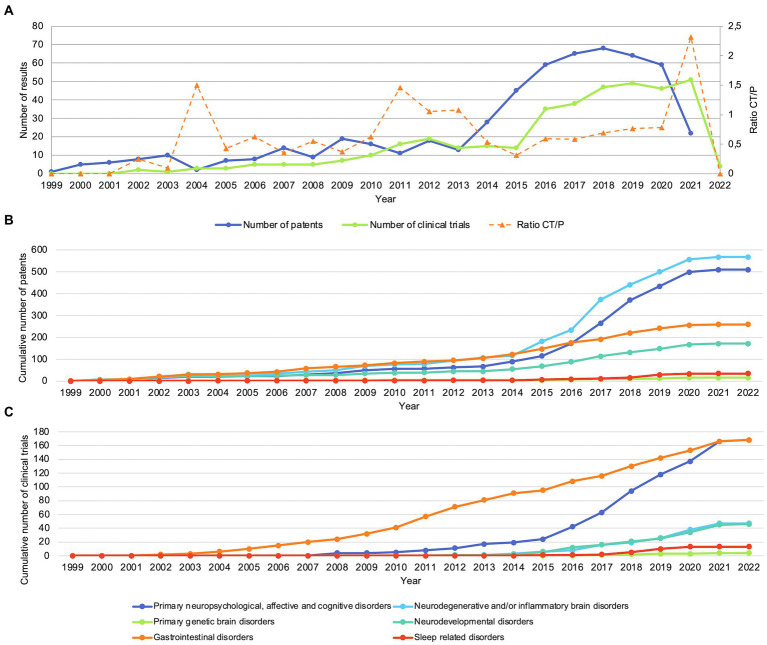
Patents and clinical trials show an increase in numbers from approximately 2013. **(A)** Number of patents and clinical trials per year. CT/P: ratio of clinical trials and patents. **(B)** Cumulative number of patents per indication group. **(C)** Cumulative number of clinical trials per indication group.

As shown in Figure 3A, the numbers of patents and clinical trials per year showed a slight upward trend between 1999 and 2013, with a steep increase from 2013 onwards. The trend of clinical trials seemed to lag approximately 2 years behind that of patents, with the first clinical trials starting in 2001 compared to the first patents being applied for in 1999. Figure 3B shows the cumulative number of patents per year for the mental, neurological and gastrointestinal indication groups. The differences in growth gradually increased over time, with neuropsychological, affective and cognitive disorders and neurodegenerative and/or inflammatory brain disorders showing the highest increase, followed by gastrointestinal disorders and neurodevelopmental disorders. Lastly, the number of patents related to primary genetic brain disorders and sleep related disorders remained the lowest. Figure 3C shows similar trends for the cumulative numbers of clinical trials per year for primary genetic brain disorders, sleep related disorders, neurodevelopmental disorders, and primary neuropsychological, affective and cognitive disorders. However, compared with the cumulative number of patents, clinical trials related to gastrointestinal and neurodegenerative disorders showed a higher growth while the number of clinical trials related to inflammatory brain disorders remained relatively low.

**Figure 4:**
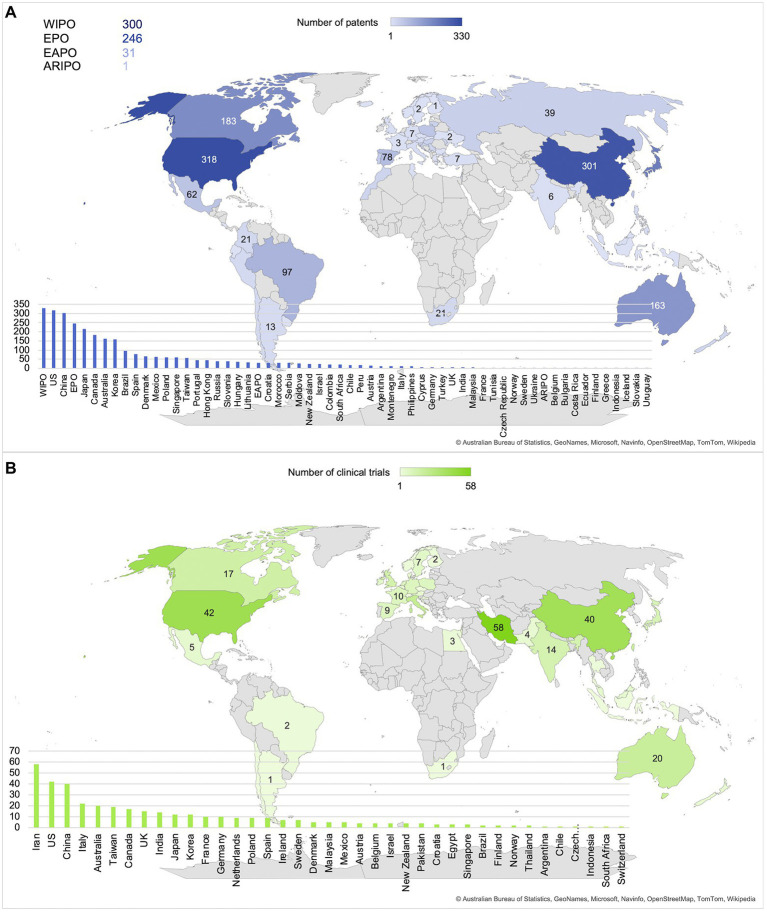
Main locations of patents and clinical trials are the United States, China, and Iran. **(A)** Number of patents per country or organization. **(B)** Number of clinical trials per country.

In Figure 4, maps are displayed to represent the number of patents that are applied for (Figure 4A) and the number of clinical trials that are executed (Figure 4B) in each country. Disregarding patents that were applied for at regional patent organizations such as the World Intellectual Property Organization (WIPO; *n* = 300), Euopean Patent Office (EPO; *n* = 246), Eurasian Patent Organization (EAPO; *n* = 31) and African Regional Intellectual Property Organization (ARIPO; *n* =1), the main countries for both patents and clinical trials included the US (*n* = 318 and *n* = 42) and China (*n* = 301 and *n* = 40), with the addition of Iran being the country where the highest number of clinical trials were executed (*n* = 58). Overall, Asia, North America and Australia appear to be the main patent and clinical trial locations.

### Applicant and sponsor characteristics

**Figure 5:**
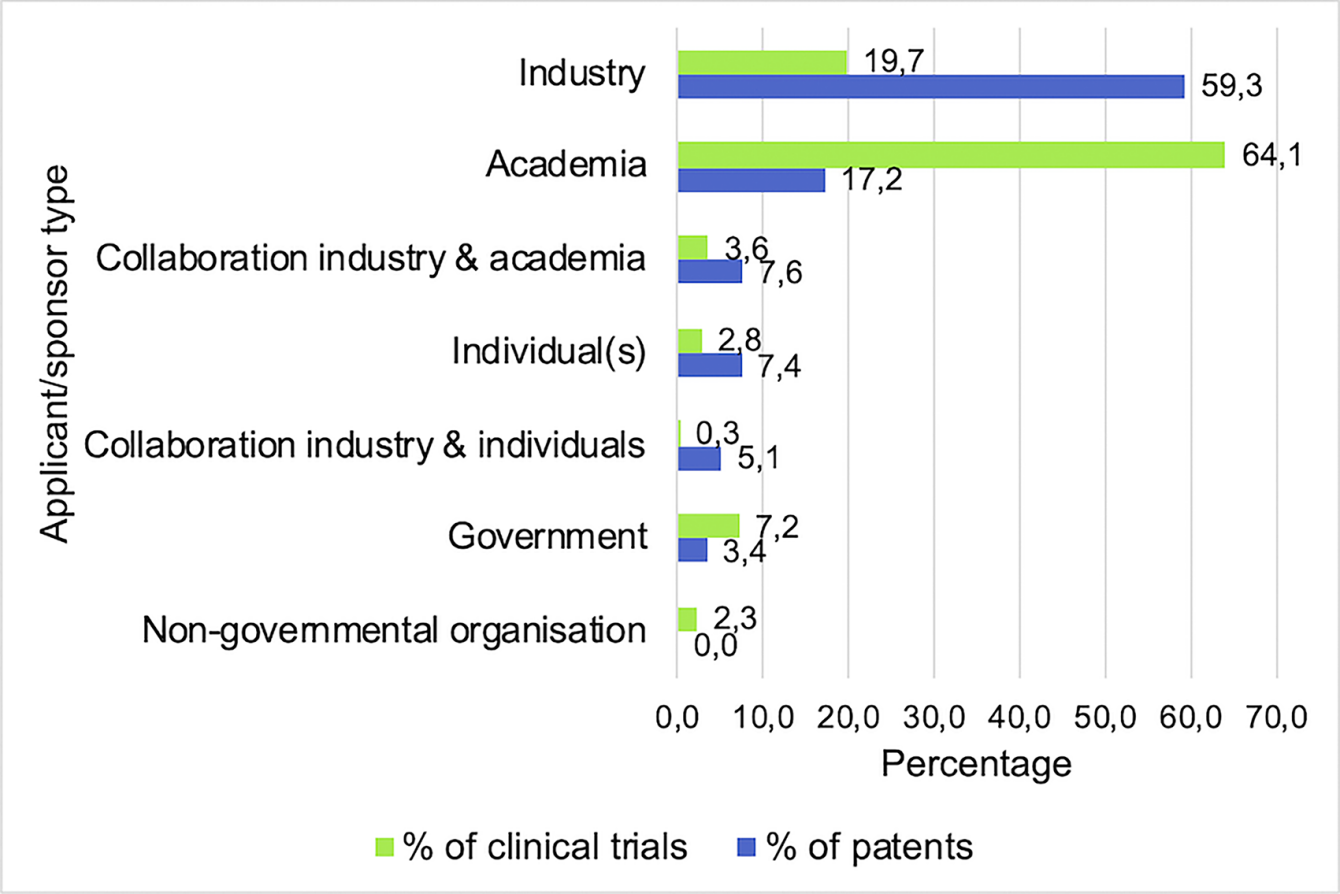
Main applicant/sponsor types are industrial and academic. Bars indicate the percentages of all clinical trials and patents sponsored or applied by each different type of entity.

Finally, Figure 5 shows the proportion (%) of different applicant or sponsor types that contributed to the patents and clinical trials included in this study. The main patent applicant types consisted of industry (59.3%) and academia (17.2%), followed by collaborations between the two (7.6%) and individuals (7.4%). For clinical trials, the main sponsors were academia (64.1%) and industry (19.7%), followed by governmental sponsors (7.2%).

## Discussion

The current study provides an overview of the state of the art of probiotics as possible clinical intervention modalities for BGMA related indications. In total, 565 patents and 390 clinical trials were investigated, showing an overall increase in number over the past ~20 years with a focus on neuropsychological, affective and cognitive disorders, neurodegenerative and/or inflammatory disorders, and gastrointestinal disorders. The main locations for patent applications were the US and China, and clinical trials were mainly executed in Iran.

Considering the overall development of the field over time, the numbers of patents and clinical trials begin to increase significantly around 2013. A simple search in PubMed using the keywords “probiotics,” “gut” and “brain” reveals that this trend also applies to the number of scientific publications. Moreover, the increase coincides with the publication of a popular review on this topic (Cryan and Dinan, 2012), which may have contributed to the high overall increase in research on BGMA-associated disorders. More specifically, a distinction can be seen between the different groups of indications included in this study, with neuropsychological, affective and cognitive disorders, neurodegenerative and/or inflammatory disorders, gastrointestinal disorders and neurodevelopmental disorders showing more growth compared to primary genetic brain disorders and sleep related disorders. This is not surprising, as genetic brain disorders are relatively rare and burdensome, requiring highly complex research and treatments (Uhl and Grow, 2004).

Additionally, it was observed that the trend of the number of clinical trials per year lagged approximately 2 years behind that of patents. A similar trend was found in a previous study comparing patents and clinical trials (Wiegers et al., 2022). This result could be expected, as clinical trials are a later step in the research process than patents (van de Burgwal et al., 2018). To clarify, patents are intended to protect new ideas and products whereas clinical trials can be considered tools for further development and testing of a product. This also relates to the fact that the distribution of patents and clinical trials differed between indications. For example, even though the total sum of patents included in this study was higher than that of clinical trials, it was found that compared to patents, a higher number of clinical trials was related to indications such as cognition, constipation, and abdominal pain. A possible explanation could be that, for these indications in particular, it is easier to measure certain variables through biomarkers in order to demonstrate an effect of probiotics. According to van den Nieuwboer et al. (2016), one of the main barriers in probiotics research is the lack of biomarkers to properly assess and demonstrate the efficacy of probiotics as clinical intervention modalities.

Regarding the locations of probiotic research, both patents and clinical trials were predominantly applied for or carried out in North America and Asia. While patents and clinical trials show a similar pattern regarding overall global distribution, one large difference is that many clinical trials were located in Iran. Further investigation of this phenomenon showed that it is in accordance with previous research, stating that probiotic research on the BGMA is growing exponentially in Iran and China (Dronkers et al., 2020). However, this does not explain the complete lack of patents applied for in Iran. A possible contributing factor is the differentiation between probiotic research and industry. According to Damari et al. (2021), a negative growth of the probiotic market was observed in Iran between 2009 and 2014. In contrast to China, one of the countries with the highest numbers of patents and clinical trials, as well as an expected market growth of 9.8% in the years following 2020 (Globe Newswire, 2020), it may be possible that the lack of patents applied for in Iran is caused by a lack of market interest. A closer look at the clinical trial data that were collected reveals that all clinical trials executed in Iran were sponsored by academia, which is in accordance with the finding that patents were mainly applied for by the industry and clinical trials were mainly sponsored or executed by academia. This is a likely outcome because patent applications can be costly and time consuming, and therefore are usually done with the expectations of profits in the long run (van de Burgwal et al., 2018). Clinical trials on the other hand are usually executed to test the safety and efficacy of a product on a shorter term, with a higher focus on acquiring scientific knowledge instead of profits (Janse et al., 2020).

Comparing the findings of this study with previous research on the potential of probiotics as clinical intervention modalities for infectious diseases (Wiegers et al., 2022), similarities were found regarding the locations of patents and clinical trials, as well as the applicant and sponsor types. The development of the fields over time appeared to differ slightly, with patents and clinical trials focused on infectious diseases showing a more gradual growth since the start of the 21st century, while patents and clinical trials focused on BGMA related indications showed a more exponential growth starting around 2013. A possible explanation for this may be the fact that infectious diseases have been considered an important public health issue for longer than mental and neurological indications, together with the BGMA being a relatively new concept in its entirety.

When interpreting the results of this review, some limitations need to be considered. Firstly, this review was focused on BGMA-associated indications. The term BGMA is relatively new, and there are no criteria set to determine whether a condition is considered BGMA related or not. Therefore, as many brain disorders as possible were included in order to minimize the risk of missing important results. For digestive tract disorders, only results that were related to any type of brain indications were included. Secondly, the clinical trial records included in this study were selected based on their focus and setup, regardless of status. This may cause bias, as some of the clinical trials may not have been completed at the time of analysis. However, with the aim of providing insight into the total research landscape in mind, this was considered preferable over the inclusion of only clinical trials with published results. Thirdly, the trends of the numbers of patents and clinical trials during the years 2020 to 2022 may lack accuracy due to the COVID-19 pandemic as well as the 18-month patent application process. Still, the broad scope of this review together with the thorough analysis of patents and clinical trials have resulted in a useful overview of the development of the research field.

To conclude, the state of the art of probiotics as clinical intervention modality is showing an increasing interest from the industry as reflected by the number of registered patents, as well as from academia, reflected by registration of clinical trials. The increasing societal and personal burden of mental and neurological disorders can be considered a call to action. Future research should elucidate which types of probiotics have the highest potential to lower this burden in a cost-effective manner. Finally, the inextricable link between the brain, gut and microbiota demonstrates the importance of a more holistic approach to the treatment of not only infectious diseases, but also non-communicable diseases of mental, neurological and gastrointestinal nature.

## Author contributions

CW analysis and writing. MV analysis and writing first draft. RB reviewing and editing. OL conceptualization, reviewing and editing. All authors contributed to the article and approved the submitted version.

## Funding

The contribution of CW is financed by Yakult Nederland B.V. The funder was not involved in the study design, collection, analysis, interpretation of data, the writing of this article, or the decision to submit it for publication.

## Conflict of interest

RB is also a member of the Scientific Advisory Board of Chr Hansen A/S., OL is also Senior Manager Science at Yakult Nederland B.V.

The remaining authors declare that the research was conducted in the absence of any commercial or financial relationships that could be construed as a potential conflict of interest.

## Publisher’s note

All claims expressed in this article are solely those of the authors and do not necessarily represent those of their affiliated organizations, or those of the publisher, the editors and the reviewers. Any product that may be evaluated in this article, or claim that may be made by its manufacturer, is not guaranteed or endorsed by the publisher.

## References

[ref1] American Psychiatric Association. (2013). Diagnostic and Statistical Manual of Mental Disorders. (5th Edn.) Available at: 10.1176/appi.books.9780890425596 (Accessed March 10, 2022).

[ref2] Ansari F. Pourjafar H. Tabrizi A. Homayouni A. (2020). The effects of probiotics and prebiotics on mental disorders: a review on depression, anxiety, Alzheimer, and autism Spectrum disorders. Curr. Pharm. Biotechnol. 21, 555–565. doi: 10.2174/1389201021666200107113812, PMID: 31914909

[ref3] Arneth B. M. (2018). Gut–brain axis biochemical signalling from the gastrointestinal tract to the central nervous system: gut dysbiosis and altered brain function. Postgrad. Med. J. 94, 446–452. doi: 10.1136/postgradmedj-2017-135424, PMID: 30026389

[ref4] Bercik P. Verdu E. F. Foster J. A. Macri J. Potter M. Huang X.. (2010). Chronic gastrointestinal inflammation induces anxiety-like behavior and alters central nervous system biochemistry in mice. Gastroenterology 139, 2102–2112.e1. doi: 10.1053/j.gastro.2010.06.063, PMID: 20600016

[ref5] Chang L. Wei Y. Hashimoto K. (2022). Brain-gut-microbiota axis in depression: a historical overview and future directions. Brain Res. Bull. 182, 44–56. doi: 10.1016/j.brainresbull.2022.02.004, PMID: 35151796

[ref6] Chong P. P. Chin V. K. Looi C. Y. Wong W. F. Madhavan P. Yong V. C. (2019). The microbiome and irritable bowel syndrome–a review on the pathophysiology, current research and future therapy. Front. Microbiol. 10:1136. doi: 10.3389/fmicb.2019.01136, PMID: 31244784 PMC6579922

[ref7] ClinicalTrials.gov. (2021). ClinicalTrials.Gov background. Available at: https://clinicaltrials.gov/ct2/about-site/background (Accessed March 9, 2022).

[ref8] Cryan J. F. Dinan T. G. (2012). Mind-altering microorganisms: the impact of the gut microbiota on brain and behaviour. Nat. Rev. Neurosci. 13, 701–712. doi: 10.1038/nrn3346, PMID: 22968153

[ref9] Damari B. Tajabadi Ebrahimi M. Jafarvand E. (2021). Probiotics industry: review of current challenges and the future roadmap. Payesh 20, 263–273. doi: 10.52547/payesh.20.3.263

[ref10] Dronkers T. M. Ouwehand A. C. Rijkers G. T. (2020). Global analysis of clinical trials with probiotics. Heliyon 6:e04467. doi: 10.1016/j.heliyon.2020.e04467, PMID: 32715136 PMC7371762

[ref11] Duke University Health System. (2022). Autoimmune Brain Diseases. Available at: https://www.dukehealth.org/pediatric-treatments/autoimmune-brain-disorders (Accessed March 10, 2022).

[ref12] Edebol Carlman H. M. Rode J. König J. Repsilber D. Hutchinson A. N. Thunberg P.. (2022). Probiotic mixture containing lactobacillus helveticus, *Bifidobacterium longum* and *Lactiplantibacillus plantarum* affects brain responses to an arithmetic stress task in healthy subjects: a randomised clinical trial and proof-of-concept study. Nutrients 14:1329. doi: 10.3390/nu14071329, PMID: 35405944 PMC9002567

[ref13] Espacenet. (2019a). Classification Search. Available at: https://worldwide.espacenet.com/classification?locale=en_EP (Accessed September 19, 2022).

[ref14] Espacenet. (2019b). What is the Cooperative Patent Classification System? Available at: https://worldwide.espacenet.com/help?quickHelpPage=classificationsearchCPC.1&locale=en_EP&method=handleQuickHelp (Accessed October 28, 2022).

[ref15] European Patent Office (2022). Espacenet Patent Search. Available at: https://www.epo.org/searching-for-patents/technical/espacenet.html (Accessed July 12, 2022).

[ref16] Feddema J. J. Claassen E. (2018). Addressing the unmet need in respiratory viruses: an interdisciplinary analysis of product development pipeline in Asia. Int. J. Clin. Trials 5, 1–10. doi: 10.18203/2349-3259.ijct20180001

[ref17] Fijan S. (2014). Microorganisms with claimed probiotic properties: an overview of recent literature. Int. J. Environ. Res. Public Health 11, 4745–4767. doi: 10.3390/ijerph110504745, PMID: 24859749 PMC4053917

[ref18] Globe Newswire (2020). Probiotics Market Worldwide is Projected to Grow by US$27.4 Billion. Available at: https://www.globenewswire.com/news-release/2020/05/22/2037833/0/en/Probiotics-market-worldwide-is-projected-to-grow-by-US-27-4-Billion.html (Accessed July 12, 2022).

[ref19] Góralczyk-Bińkowska A. Szmajda-Krygier D. Kozłowska E. (2022). The microbiota–gut–brain Axis in psychiatric disorders. Int. J. Mol. Sci. 23:11245. doi: 10.3390/ijms231911245, PMID: 36232548 PMC9570195

[ref20] Hariton E. Locascio J. J. (2018). Randomised controlled trials—the gold standard for effectiveness research. BJOG 125:1716. doi: 10.1111/1471-0528.15199, PMID: 29916205 PMC6235704

[ref21] Hill C. Guarner F. Reid G. Gibson G. R. Merenstein D. J. Pot B.. (2014). The international scientific Association for Probiotics and Prebiotics consensus statement on the scope and appropriate use of the term probiotic. Nat. Rev. Gastroenterol. Hepatol. 11, 506–514. doi: 10.1038/nrgastro.2014.66, PMID: 24912386

[ref22] Hou Y. Dan X. Babbar M. Wei Y. Hasselbalch S. G. Croteau D. L.. (2019). Ageing as a risk factor for neurodegenerative disease. Nat. Rev. Neurol. 15, 565–581. doi: 10.1038/s41582-019-0244-7 31501588

[ref23] Hutchinson A. N. Tingö L. Brummer R. J. (2020). The potential effects of probiotics and ω-3 fatty acids on chronic low-grade inflammation. Nutrients 12:2402. doi: 10.3390/nu12082402, PMID: 32796608 PMC7468753

[ref24] Janse M. J. Trocha M. Feddema J. Claassen E. Van de Burgwal L. (2020). Identifying the gaps in human and veterinary chlamydia vaccine development. Int. J. Clin. Trials 7, 160–169. doi: 10.18203/2349-3259.ijct20203102

[ref25] Kim Y. K. Shin C. (2018). The microbiota-gut-brain axis in neuropsychiatric disorders: pathophysiological mechanisms and novel treatments. Curr. Neuropharmacol. 16, 559–573. doi: 10.2174/1570159X15666170915141036, PMID: 28925886 PMC5997867

[ref800] Larsen O. F. van de Burgwal L. H. (2021). On the verge of a catastrophic collapse? The need for a multi-ecosystem approach to microbiome studies. Front. Microbiol. 12:784797. doi: 10.3389/fmicb.2021.784797, PMID: 34925292 PMC8674555

[ref26] Liu L. Zhu G. (2018). Gut–brain axis and mood disorder. Front. Psych. 9:223. doi: 10.3389/fpsyt.2018.00223, PMID: 29896129 PMC5987167

[ref27] Martin C. R. Osadchiy V. Kalani A. Mayer E. A. (2018). The brain-gut-microbiome Axis. Cell. Mol. Gastroenterol. Hepatol. 6, 133–148. doi: 10.1016/j.jcmgh.2018.04.003, PMID: 30023410 PMC6047317

[ref28] Morais L. H. Schreiber H. L. Mazmanian S. K. (2021). The gut microbiota–brain axis in behaviour and brain disorders. Nat. Rev. Microbiol. 19, 241–255. doi: 10.1038/s41579-020-00460-0, PMID: 33093662

[ref29] Neevel A. M. Urias E. Claassen E. van de Burgwal L. H. (2020). Quantity vs. quality: an assessment of the current pipeline for rabies. Trop. Med. Int. Health 25, 397–407. doi: 10.1111/tmi.13367, PMID: 31872495

[ref30] Palepu M. S. K. Dandekar M. P. (2022). Remodeling of microbiota gut-brain axis using psychobiotics in depression. Eur. J. Pharmacol. 931:175171. doi: 10.1016/j.ejphar.2022.175171, PMID: 35926568

[ref31] Ramezanpour B. Riemens T. van de Burgwal L. H. M. Claassen E. (2015). An interdisciplinairy analysis of genetically modified vaccines: from clinical trials to market. Int. J. Clin. Trials 2, 64–74. doi: 10.18203/2349-3259.ijct20151235

[ref32] Rode J. Carlman H. M. E. König J. Repsilber D. Hutchinson A. N. Thunberg P.. (2022). Probiotic mixture containing lactobacillus helveticus, *Bifidobacterium longum* and *Lactiplantibacillus plantarum* affects brain responses toward an emotional task in healthy subjects: a randomized clinical trial. Front. Nutr. 9, 1–15. doi: 10.3389/fnut.2022.827182, PMID: 35571902 PMC9104811

[ref33] Sampson T. R. Debelius J. W. Thron T. Janssen S. Shastri G. G. Ilhan Z. E.. (2016). Gut microbiota regulate motor deficits and neuroinflammation in a model of Parkinson’s disease. Cells 167, 1469–1480.e12. doi: 10.1016/j.cell.2016.11.018, PMID: 27912057 PMC5718049

[ref34] Sanmarco L. M. Wheeler M. A. Gutiérrez-Vázquez C. Polonio C. M. Linnerbauer M. Pinho-Ribeiro F. A.. (2021). Gut-licensed IFNγ+ NK cells drive LAMP1+ TRAIL+ anti-inflammatory astrocytes. Nature 590, 473–479. doi: 10.1038/s41586-020-03116-4, PMID: 33408417 PMC8039910

[ref35] Sarkar A. Lehto S. M. Harty S. Dinan T. G. Cryan J. F. Burnet P. (2016). Psychobiotics and the manipulation of bacteria-gut-brain signals. Trends Neurosci. 39, 763–781. doi: 10.1016/j.tins.2016.09.002, PMID: 27793434 PMC5102282

[ref36] Silva Y. P. Bernardi A. Frozza R. L. (2020). The role of short-chain fatty acids from gut microbiota in gut-brain communication. Front. Endocrinol. 11:25. doi: 10.3389/fendo.2020.00025, PMID: 32082260 PMC7005631

[ref37] Skrzydło-Radomańska B. Prozorow-Król B. Cichoż-Lach H. Majsiak E. Bierła J. B. Kanarek E.. (2021). The effectiveness and safety of multi-strain probiotic preparation in patients with diarrhea-predominant irritable bowel syndrome: a randomized controlled study. Nutrients 13:756. doi: 10.3390/nu13030756, PMID: 33652763 PMC7996889

[ref38] Suganya K. Koo B. S. (2020). Gut–brain Axis: role of gut microbiota on neurological disorders and how probiotics/prebiotics beneficially modulate microbial and immune pathways to improve brain functions. Int. J. Mol. Sci. 21:7551. doi: 10.3390/ijms21207551, PMID: 33066156 PMC7589356

[ref39] Thorpe K. E. Levey A. I. Thomas J. (2021). U.S. Burden of Neurodegenerative Disease. Available at: https://www.fightchronicdisease.org/sites/default/files/May%202021%20Neurodegenerative%20Disease%20Burden%20on%20US%20-%20FINAL%20.pdf (Accessed July 14, 2022).

[ref40] Uhl G. R. Grow R. W. (2004). The burden of complex genetics in brain disorders. Arch. Gen. Psychiatry 61, 223–229. doi: 10.1001/archpsyc.61.3.223 14993109

[ref41] Umbrello G. Esposito S. (2016). Microbiota and neurologic diseases: potential effects of probiotics. J. Transl. Med. 14, 298–211. doi: 10.1186/s12967-016-1058-7, PMID: 27756430 PMC5069982

[ref42] US National Library of Medicine (2016). Genetic Brain Disorders. MedlinePlus. Available at: https://medlineplus.gov/geneticbraindisorders.html (Accessed March 10, 2022).

[ref43] US National Library of Medicine (2021). Degenerative Nerve Diseases. MedlinePlus. Available at: https://medlineplus.gov/degenerativenervediseases.html (Accessed March 10, 2022).

[ref44] van de Burgwal L. H. M. van der Waal M. B. Claassen E. (2018). Accelerating microbiota product development: the societal impact value cycle as a conceptual model to shape and improve public-private valorization processes. PharmaNutrition 6, 157–168. doi: 10.1016/j.phanu.2018.07.002

[ref45] van den Nieuwboer M. van de Burgwal L. H. M. Claassen E. (2016). A quantitative key-opinion-leader analysis of innovation barriers in probiotic research and development: valorisation and improving the tech transfer cycle. PharmaNutrition 4, 9–18. doi: 10.1016/j.phanu.2015.09.003

[ref46] van der Geest A. M. Schukking I. Brummer R. Pieterse H. van den Nieuwboer M. van de Burgwal L.. (2022b). Inadequate safety reporting in the publications of randomised clinical trials in irritable bowel syndrome: drug versus probiotic interventions. Benef. Microbes 13, 195–204. doi: 10.3920/BM2021.0124, PMID: 35848114

[ref47] van der Geest A. M. Schukking I. Brummer R. van de Burgwal L. Larsen O. (2022a). Comparing probiotic and drug interventions in irritable bowel syndrome: a meta-analysis of randomised controlled trials. Benef. Microbes 13, 183–194. doi: 10.3920/BM2021.0123, PMID: 35848115

[ref48] Villumsen M. Aznar S. Pakkenberg B. Jess T. Brudek T. (2019). Inflammatory bowel disease increases the risk of Parkinson’s disease: a Danish nationwide cohort study 1977–2014. Gut 68, 18–24. doi: 10.1136/gutjnl-2017-315666, PMID: 29785965

[ref49] Wiegers C. Van de Burgwal L. H. Larsen O. F. (2022). Probiotics for the management of infectious diseases: reviewing the state of the art. Front. Microbiol. 13, 1–11. doi: 10.3389/fmicb.2022.877142, PMID: 35572661 PMC9096241

[ref50] World Health Organization (2021a). Adolescent Mental Health. Available at: https://www.who.int/news-room/fact-sheets/detail/adolescent-mental-health (Accessed July 11, 2022).

[ref51] World Health Organization (2021b). Mental Health Investment Case: A Guidance Note. Available at: https://apps.who.int/iris/bitstream/handle/10665/340246/9789240019386-eng.pdf?sequence=1 (Accessed July 14, 2022).

[ref52] World Health Organization (2021c). Data Providers. ICTRP Registry Network. Available at: https://www.who.int/clinical-trials-registry-platform/network/data-providers (Accessed March 9, 2022).

[ref53] Zhang B. Wang H. E. Bai Y. M. Tsai S. J. Su T. P. Chen T. J.. (2021). Inflammatory bowel disease is associated with higher dementia risk: a nationwide longitudinal study. Gut 70, 85–91. doi: 10.1136/gutjnl-2020-320789, PMID: 32576641

