# Supplementary material for: Reviewing the state of the art of probiotics as clinical modalities for brain–gut–microbiota axis associated disorders
Source: Front Microbiol. 2022 Nov 25;13:1053958. doi: 10.3389/fmicb.2022.1053958 (PMC9732675; doi:10.3389/fmicb.2022.1053958)
Supplement: Supplementary file 1 [file Data_Sheet_1.docx]

Supplementary Material


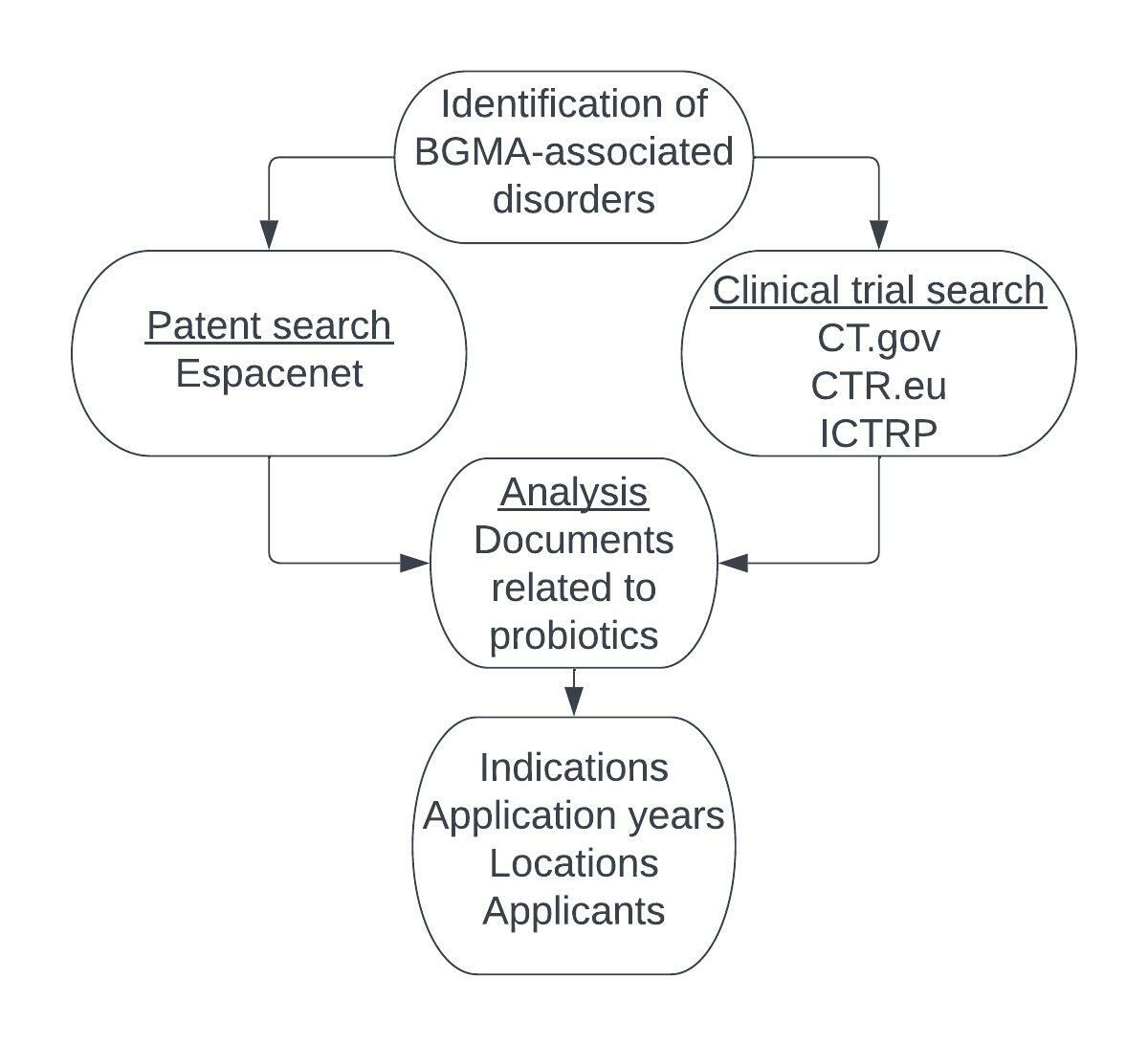


**Supplementary Figure 1. Schematic overview of the workflow**. BGMA = brain-gut-microbiota axis. CT.gov = ClinicalTrials.gov. CTR.eu = EU Clinical Trials Registry. ICTRP = International Clinical Trials Registry Platform.

Supplementary Table 1. Classification of mental, neurological and gastrointestinal indications

| **Primary neuropsychological, affective and cognitive disorders** | **Neurodegenerative and/or inflammatory brain disorders** | **Primary genetic brain disorders** | **Neurodevelopmental disorders** | **Gastrointestinal disorders** | **Sleep related disorders** |
| --- | --- | --- | --- | --- | --- |
| Acute stress disorder | Alper's disease | Aicardi syndrome | Asperger's | Abdominal pain | Breathing related sleep disorders |
| Adjustment disorders | Alzheimer's disease | Fabry disease | Attention deficit disorder | Centrally mediated abdominal pain syndrome | Insomnia disorder |
| Agoraphobia | Amyotropic Lateral Sclerosis | Fahr's syndrome | Attention-Deficit/Hyperactivity Disorder | Constipation | Narcolepsy |
| Alcohol-related disorders | Autoimmune encephalitis | Lesch-Nyhan syndrome | Autism Spectrum Disorder | Functional bowel problems | Sleep |
| Anorexia nervosa | Autoimmune-related epilepsy | Leukodystrophies | Communication disorders | Functional dyspepsia |  |
| Antisocial personality disorder | Batten disease | Menkes disease | Intellectual disabilities | Irritable Bowel Syndrome |  |
| Anxiety | Cerebral lupus | Moyamoya disease | Learning disorder |  |  |
| Avoidant/restrictive food intake disorder | Cerebro-oculo-skeletal syndrome | Niemann-Pick disease | Motor disorders |  |  |
| Binge-eating disorder | CNS vasculitis | Phenylketonuria | Palsy |  |  |
| Bipolar disorder | Corticobasal degeneration | Prader-Willi | Selective mutism |  |  |
| Body dysmorphic disorder | Friedreich ataxia | RETT syndrome | Tourette's syndrome |  |  |
| Brief psychotic disorder | Gerstmann-Straussler-Scheinker disease | Tay-Sachs disease |  |  |  |
| Bulimia nervosa | Hashimoto's disease | Wilson disease |  |  |  |
| Caffeine-related disorders | Huntington's disease | Zellweger syndrome |  |  |  |
| Cannabis-related disorders | Leigh's disease |  |  |  |  |
| Catatonia | Lewy body disease |  |  |  |  |
| Cognition | Monomyelic amyotrophy |  |  |  |  |
| Conduct disorder | Multiple Sclerosis |  |  |  |  |
| Conversion disorder | Multiple system atrophy |  |  |  |  |
| Cyclothymic disorder | Neuro-Behcet's disease |  |  |  |  |
| Delirium | Neurodegeneration with brain iron accumulation |  |  |  |  |
| Depersonalization/derealization disorder | Neuromyelitis optica |  |  |  |  |
| Depression | Neurosarcoidosis |  |  |  |  |
| Disinhibited social engagement disorder | Opsoclonus myoclonus |  |  |  |  |
| Disruptive mood dysregulation disorder | Optic neuritis |  |  |  |  |
| Dissociative amnesia | Parkinson's disease |  |  |  |  |
| Dissociative identity disorder | Pick's disease |  |  |  |  |
| Excoriation disorder | Prion diseases |  |  |  |  |
| Factitious disorder | Progressive multifocal leukoencephalopathy |  |  |  |  |
| General personality disorders | Spinal muscular atrophy |  |  |  |  |
| Generalized anxiety disorder | Striatonigral degeneration |  |  |  |  |
| Hallucinogen-related disorders |  |  |  |  |  |
| Hoarding disorder |  |  |  |  |  |
| Hypersomnolence disorder |  |  |  |  |  |
| Illness anxiety disorder |  |  |  |  |  |
| Impulsivity |  |  |  |  |  |
| Inhalant-related disorders |  |  |  |  |  |
| Intermittent explosive disoder |  |  |  |  |  |
| Kleptomania |  |  |  |  |  |
| Major and mild neurocognitive disorders |  |  |  |  |  |
| Major depressive disorder, single and recurrent |  |  |  |  |  |
| Mania |  |  |  |  |  |
| Migraine |  |  |  |  |  |
| Mood |  |  |  |  |  |
| Obsessive-Compulsive Disorder |  |  |  |  |  |
| Opioid-related disorders |  |  |  |  |  |
| Oppositional defiant disorder |  |  |  |  |  |
| Other neurodevelopmental disorders |  |  |  |  |  |
| Panic attack |  |  |  |  |  |
| Panic disorder |  |  |  |  |  |
| Parasomnias |  |  |  |  |  |
| Persistent depressive disorder |  |  |  |  |  |
| Pica |  |  |  |  |  |
| Posttraumatic Stress Disorder |  |  |  |  |  |
| Premenstrual dysphoric disorder |  |  |  |  |  |
| Pyromania |  |  |  |  |  |
| Reactive attachment disorder |  |  |  |  |  |
| Rumination disorder |  |  |  |  |  |
| Schizoaffective disorder |  |  |  |  |  |
| Schizophrenia |  |  |  |  |  |
| Schizotypal (personality) disorder |  |  |  |  |  |
| Sedative-, hypnotic-, or anxiolytic-related disorders |  |  |  |  |  |
| Separation anxiety disorder |  |  |  |  |  |
| Social anxiety disorder |  |  |  |  |  |
| Somatic symptom disorder |  |  |  |  |  |
| Specific phobia |  |  |  |  |  |
| Stimulant-related disorders |  |  |  |  |  |
| Stress |  |  |  |  |  |
| Substance-related disorders |  |  |  |  |  |
| Tobacco-related disorders |  |  |  |  |  |
| Trichotillomania |  |  |  |  |  |
